# 

**Published:** 1887-11-19

**Authors:** 


### Notice to Advertisers.

The scale of charges for small prepaid Advertisements—Situations Wanted, Situations Vacant, &c., &c., is as follows:—

| | s. | d. |
|---|---|---|
| One insertion of three lines | 1 | 0 |
| Three " " " | 2 | 6 |
| Per line afterwards | 0 | 6 |

*A line averages ten words.*

All copy for Advertisements must be sent to A. P. Watt, 2, Paternoster Square, E.C., by first post on the Tuesday *preceding* publication.

### NOTICE TO CORRESPONDENTS.

All correspondence must be written on one side of the paper only.

We cannot undertake to return MSS. not used.

**Communications,** whether intended for insertion or for private information, must be authenticated by the names and addresses of the writers, not necessarily for publication.

**Local papers** containing reports or news paragraphs **should be marked.**

It is especially requested that early intelligence of **local events of interest,** or which it may be thought desirable to bring under notice, may be sent direct to the Editor.

**All communications respecting editorial matters** should be addressed to the Editor, Norfolk House, Norfolk Street, Strand, London, W.C.

### Amusements with Profit for all Ages.

**Rules.**—All answers must be addressed to the Prize Editor, The Hospital, Norfolk House, Norfolk Street, Strand, W.C., not later than the *first* post on Friday next. The full name and address must in all cases be given, but a *nom de plume* may be added for publication. Only one side of the paper must be written on.

No one will be permitted to win the Monthly or Quarterly Prizes twice in any one year, the annual prizes being offered especially to prevent this.

### FOR MEN AND WOMEN.

### I.—The Quilter Prizes. Six Guineas.

Mr. Cuthbert Quilter, M.P., has placed certain funds at our disposal, as already announced, some of which we shall expend upon prizes in this column. Prizes will be given

#### For best Specimens of Work.

Three prizes of three guineas, two guineas, and one guinea, will be given to the competitor who sends in the first, second, and third best specimens of work of any kind, suitable for presentation to the inmates of the Hospitals, addressed to the Editor, The Lodge, Porchester Square, W., on or before the 31st of December next. This work may take the form of a toy, a doll, or a scrap book for the children; an article of clothing, a painting or watercolour, an illuminated card or any other gift, for men or women, or for to aid decoration. It is proposed to arrange for the distribution of the articles sent in for this competition amongst the patients in the hospitals.

### II.—Word and Literary Competition.

Commenced November 5th, 1887; ends January 28th, 1888.

Three prizes of one guinea, 15*s.* and 10*s.* 6*d.* will be given each quarter to the three competitors who obtain the highest number of marks: (1) by making the largest number of words derived from the word or phrase set for anatomical dissection; or (2) for the best answers to (6); or (3) by (1) and (2) combined.

(A) THE WORD COMPETITION.

We shall give the newspapers for disection during the first quarter, that for this week being

"The Illustrated London News."

Observe.—Proper names, abbreviations, foreign words, words of less than four letters and repetitions are barred. Nuttall's dictionary only to be used.

(B) THE LITERARY COMPETITION.

Some people hate puzzles of all kinds, including word competitions, but they are fond of corre spondence, and many sisters and mothers are continually receiving and sending letters to and from members of the family who are abroad in India or the Colonies. The constant coming and going between England and other portions of the British Empire make it a matter of interest and importance to know something about the life, the climate, the amusements, the adventures, the clothing, and the surroundings of those who seek a livelihood beyond the seas. We shall therefore try to find space for the publication of selected letters from abroad, which may be sent by any of our readers who have friends and relations in foreign lands. We shall give marks for these letters as well as for the word competitions which will count equally for these prizes, so that anyone may enter for both or either, the prizes to be given to those who get the highest number of marks. The first letter from across the seas will be found on page 93 of The Hospital for Nov. 5th last. It is a record of adventure in Australia, and gives a good idea of one class of letter suitable for this column.

#### The Word Competition—Results of First Week.

"The Times."

*Accepted Scores.*—Ellice (51), L'arc en ciel (50), Reldas (47), Dormouse (47), Gretta (45), Tim (45), E.E. (42), Ostrich (41), Nogrub (41), Condorcet (37), "She," (35), Skye (35), Esau (35), Boss (33), Mater (33), Brownie (33), Tabs (32), Aralc (28), Kath (27), Toby (26), Lauriston (24), Puss (21).

#### Literary Competition.

*Gretta* sends a letter from India, and *Aralc* one from Canada.

Note.—In deference to the views of many readers, we are arranging an alternative to the Word Competition, to be announced next week.

#### Entertainments at Hospitals and Asylums.

We propose to give prizes of one guinea and of half-a-guinea respectively to the correspondents who send from time to time between now and the 1st of March next the best, brightest, and briefest descriptions of concerts, theatrical and other entertainments given during the winter to the inmates of any public institution.

Note.—No coupons need be sent in connection with any of these competitions, and we hope they are sufficiently varied to induce a large number of persons to enter for them. The Quilter Prizes No. I. will be open until 31st December, 1887. Competition No. II. will end on January 28th, 1888.

### THE CHILDREN'S CORNER.

#### Gunpowder Plot.

The Guy Fawkes letters this week are capital. Everyone likes the subject. I am glad to receive very good descriptions from some little defaulters, who find the exploits of "Guy Fawkes and his companions" more to their mind than the traditions of historical buildings. I hope they will "try again" now they have shown how well they *can* write, as the letters which have appeared and those which in future will be published every fortnight, prove how interesting, and even attractive, the "very dry subject of castles and cathedrals" can be made. Space does not allow of remarks this week upon the different letters, much to my regret.

From "Gem."

The Gunpowder Plot was a plot formed by the Roman Catholics, in the reign of James I., because of the severity of the laws against them. The plot was to fire a mine in a cellar under the Houses of Parliament, on 5th November, 1605, and thus to destroy the States of the realm; *i.e.*, the King, the Lords, and the Commons there assembled. The conspiracy was projected by Robert Catesby, and among the other conspirators were—Guy Faukes, who was executed in January, 1606; Percy and Catesby, who were both killed at Holbeach House, whither they had fled, 8th November, 1605; Sir Everard Digby, Rookwood, Winter, and others, who were executed on the same day as that on which Guy Faukes was. What led to the discovery of the plot was an anonymous letter to Lord Monteagle. It contained these words: "Though there be no appearance of stir, yet, I say they shall receive a terrible blow this parliament, and yet they shall not see who hurts them." King James always said that it was his cleverness that solved the riddle of this letter. However this may be, he certainly ordered some men to go and search the cellars under the House, and in among some barrels of gunpowder, covered over with sticks, they found Guy Faux guarding them. He said that if he had known of their coming he would have fired it before, and have been destroyed with it. He was a bold man, for even on the rack he would tell nobody who were his fellow-conspirators. Guy Faux cellars, where the barrels were found, stood till 1826, when they were turned into offices.

From "Skye."

Gunpowder Plot originated in the reign of James I. He was a Protestant, and the first Parliament that he summoned made and revived some severe laws against the Catholic religion. This so angered Robert Catesby, a restless Catholic gentleman of an old family, that he formed this terrible design to blow up the Parliament called the Gunpowder Plot. He confided his scheme to Thomas Winter, a Worcester gentleman, who while at Ostend found a daring Italian named Guido or Guy Fawkes, to whom he confided the secret, and brought him back to England with him. The plot was now told to two other gentlemen, Thomas Percy and John Wright. All these met in a solitary house in the open fields near London, where Catesby disclosed the full plan to them, after which they received the sacrament from a Jesuit priest called Gerrard. They hired two houses, one adjoining the Parliament, its cellars going underneath the House of Parliament, and the other on the Lambeth side of the Thames. This one they used for storing gunpowder, wood, &c., which was conveyed nightly, bit by bit, over the river to the cellars under the Parliament; and another man (Robert Kay) was admitted into the conspiracy to watch over the combustibles. They got thirty-six barrels of gunpowder into the cellar, and covered them over with coals, faggots, &c. After Parliament had been postponed several times, it was at last settled for November 5th, and as the date drew near many of the conspirators, remembering that they had friends in Parliament, felt some misgivings, and wanted to warn them, but Catesby would have them warn nobody, for it was not safe. But Tresham, one of the conspirators, had a brother-in-law (Lord Mounteagle) in the House, and he was determined he would warn him, so he sent him a mysterious letter warning him not to attend Parliament, as it would receive "a terrible blow by unknown hands." This letter was shown to the King, who guessed what it meant, but decided to let the conspirators alone until the day before the opening of Parliament. On the afternoon of the 4th Fawkes was at his post, watching, when the door was thrown open, and the Lord Chamberlain and Lord Mounteagle looked in and asked him who he was? "Oh, I am Mr. Percy's servant, looking after his fuel," he returned, upon which they went away. About three hours after, at twelve o'clock, he opened the door to look if all was quiet before he set the match, when he was instantly seized and taken before the King. Next day he was carried to the Tower, but would make no confession, even after terrible torture. On the 15th of January Fawkes and some of the others (a few of them having killed themselves) were found guilty, hanged, drawn, and quartered. Ever since this the 5th of November has been kept by boys (and girls too) with fireworks, bonfires, and a guy. The little boys here make a guy and call it a Pope, but I do not know why they call it a Pope. They carry it round, and sing a song about it for money, and then burn it at night. We made one and carried it round the garden on a chair, but it suddenly toppled over, and the body came from the legs, which were stuffed with straw. We had some fireworks at night.

*Three marks each*—Skye, Gem, Betty Burke, Poodle, Princess Ida, The Eda, Ossian. *Two marks each*—Fern, Granella.

#### Puzzles.—Third Week.

No. 9. Transpositions.

| Celebrated Generals. | Their Battles. |
| --- | --- |
| Inngllowet | Rtewlaoo |
| Looahumgrrb | Iimserla |
| Wrmcolle | Abeyns |
| Lakohvce | Pecnowar |

No. 10. Geographical Charade.

My *first*, a native of the ground,
In English counties much prevails;
My *next's* in every county found,
My *whole* was never out of Wales.

No. 11. Square Word.

. . . .
. . . .
. . . .
. . . .

1. An insect. 2. Possessed by all. 3. An ending. 4. A moveable abode.

No. 12. My *first* is an untruth, my *second* is another, and *my whole* is a flower.

#### Letters.

Next week write me a letter about the "Tower of London."

#### Results of First Week Puzzles.

No. 1. Buried Authors. Browning, Arnold, Keats.

No. 2. Because it requires more patience (patients) than any other.

No. 3. Geographical Acrostic.

C arlisl E
L isbo N
A nna N
R ig I
E phesu S

No. 4. A wig.

*All right*—Pepita, Jumps, M. S. W., Betty Burke, Poodle. *Three right*—Joe, The Young Canadian, Skye, Ossian, Gem, Plummy Fluff, A.C.K., Mount Edgecumbe, Judy, Scrooge. *Two right*—Fern, Paddy, Alsie, W. R. Mills, The Eda, Spider, A. G. S. McCulloch. *One right*—Princess Ida.

#### Notices to Correspondents.

Pepita.—Your name was printed in error for Spider, who gave Emma. But you would see that your marks were credited correctly.

Granella.—Full name, age, and address must be given. One side of the paper only to be written on.

